# Macrophage spatiotemporal plasticity in pulmonary diseases: decoding the niche at single-cell resolution

**DOI:** 10.3389/fimmu.2026.1855906

**Published:** 2026-06-18

**Authors:** Chenchen Lin, Shaohui Huang, Li Yang, Ziqi Wang, Huanhuan Zhou, Quncheng Zhang, Xiaoju Zhang

**Affiliations:** 1 Department of Respiratory and Critical Care Medicine, Zhengzhou University People’s Hospital, Henan Provincial People’s Hospital, Zhengzhou, Henan, China; 2 Henan Joint International Research Laboratory of Diagnosis and Treatment of Pulmonary Nodules, Henan Provincial People’s Hospital, Zhengzhou University People’s Hospital, Zhengzhou, Henan, China

**Keywords:** cell–cell communication, macrophage heterogeneity, metabolic reprogramming, pulmonary macrophages, single-cell RNA sequencing, spatiotemporal plasticity

## Abstract

Pulmonary gas exchange and host defense depend on the dynamic coordination of resident and recruited macrophage populations. Historically, macrophage functions have often been interpreted through the classic M1/M2 dichotomy; however, this binary framework does not capture the heterogeneity and context-dependent plasticity of macrophage states within the lung microenvironment. Advances in single-cell RNA sequencing and spatial multi-omics have substantially refined our understanding of this complex macrophage network. Here, we synthesize evidence from human studies and experimental models to summarize macrophage functional states in homeostasis and across chronic obstructive pulmonary disease, asthma, idiopathic pulmonary fibrosis, pulmonary hypertension, acute lung injury/acute respiratory distress syndrome, and lung cancer. We highlight how macrophage transcriptional programs are shaped by ontogeny, tissue niche, and epigenetic–metabolic regulation, and how these programs are linked to disease-specific remodeling of the pulmonary microenvironment. Across diverse respiratory diseases, persistent tissue injury and microenvironmental stress remodel resident macrophage programs and are frequently accompanied by the expansion and context-dependent differentiation of recruited monocyte-derived macrophages. These macrophage states are associated with inflammatory amplification, epithelial and endothelial barrier dysfunction, extracellular matrix remodeling, and tumor immune evasion. Ligand–receptor and spatial analyses further identify candidate communication axes linking macrophages with stromal, epithelial, endothelial, and immune cells, some of which appear partially conserved across disease contexts. Emerging macrophage-targeted strategies are increasingly being explored beyond broad depletion, with growing interest in context-specific reprogramming and niche modulation, including antibody-based, nanocarrier-mediated, and engineered-cell approaches. Decoding the spatiotemporal trajectories and cell–cell communication networks of specific macrophage subsets, while considering tissue context, species differences, and levels of experimental support, may help clarify mechanisms of tissue remodeling, therapeutic resistance, and macrophage-targeted intervention in complex pulmonary diseases.

## 1 Introduction

The lung serves as the primary organ for gas exchange and contains one of the largest mucosal interfaces in the human body. This unique anatomical position exposes the pulmonary parenchyma to a broad range of airborne particulates, allergens, and respiratory pathogens throughout life. As a first line of defense against exogenous stimuli, the pulmonary immune system must maintain a delicate physiological balance between effective host defense and preservation of the blood–gas barrier. In this context, macrophages, among the most abundant immune cell populations within the lung, serve as central regulators of immune surveillance, tissue homeostasis, and responses to environmental challenge (1–3).

Macrophages perform diverse biological functions within the pulmonary microenvironment. They clear pathogenic microbes, apoptotic cells, and surfactant, and also participate in antigen presentation and the initiation of adaptive immune responses. Following tissue injury, macrophages contribute to inflammation resolution and tissue repair through the secretion of cytokines and matrix-remodeling enzymes (4). This functional plasticity is shaped by both ontogeny and local microenvironmental cues. The pulmonary macrophage pool comprises tissue-resident macrophages, such as alveolar macrophages, that are established during development and maintained in part through local self-renewal, alongside macrophages recruited from peripheral blood monocytes during inflammation or tissue injury (5). Functionally, macrophages respond dynamically to local cytokines, metabolites, and tissue-derived signals through transcriptional, epigenetic, and metabolic remodeling, thereby exhibiting continuous and multidimensional phenotypic transitions across homeostatic and pathological conditions (6–9).

Faced with such a heterogeneous cell population, researchers have often used the classic M1/M2 polarization model to describe macrophage activation states in disease. Although this framework has provided a useful foundation for understanding inflammatory processes, its limitations have become increasingly apparent in complex *in vivo* microenvironments. The M1/M2 dichotomy can obscure important differences between tissue-resident and monocyte-derived macrophages, and it does not capture the continuous transcriptional evolution of macrophages within tissue niches. Furthermore, it provides limited insight into the communication networks between macrophages and surrounding epithelial, endothelial, stromal, and immune cells (10).

In recent years, single-cell RNA sequencing (scRNA-seq) and spatial multi-omics have provided powerful approaches for resolving macrophage heterogeneity beyond the limits of bulk tissue analysis (11, 12). From this perspective, this review summarizes current evidence on the spatiotemporal plasticity of pulmonary macrophages in homeostasis and disease. We first discuss the molecular foundations of macrophage phenotypes from the perspectives of ontogeny, spatial niche specialization, and epigenetic–metabolic regulation. We then examine the dynamic remodeling of macrophage states and cell–cell communication networks inferred from single-cell and spatial datasets in chronic airway inflammation, pulmonary fibrosis, pulmonary vascular disease, lung cancer, and acute lung injury. By integrating these findings, we aim to provide a conceptual framework for understanding macrophage-centered niche remodeling and for developing more precise macrophage-targeted intervention strategies.

## 2 The homeostatic landscape of pulmonary macrophages from a single-cell perspective

Under homeostatic conditions, pulmonary macrophages do not constitute a homogeneous mononuclear phagocyte population. Instead, they form a niche-organized functional landscape shaped by distinct ontogenic origins, including embryonic residency and adult monocyte derivation, as well as local tissue-specific cues. Based on anatomical localization, homeostatic pulmonary macrophages can be broadly classified into three major populations: alveolar macrophages (AMs), interstitial macrophages (IMs), and pulmonary intravascular macrophages (PIMs).

### 2.1 Alveolar macrophages: developmental origin, alveolar niche maturation, and self-renewal

In the healthy adult lung, AMs are the predominant macrophage population in the alveolar airspace. Residing within the surfactant film lining the alveoli, AMs directly encounter inhaled particulates and respiratory microbes, thereby serving as a first line of defense in the alveolar niche (13).

In mice, classical fate-mapping studies have shown that AMs are seeded during perinatal development, with fetal monocytes derived from yolk-sac erythro-myeloid progenitors representing major precursors (14, 15). After entering the developing lung and occupying the alveolar niche, these precursors acquire mature AM identity under the influence of local microenvironmental signals, particularly GM-CSF and TGF-β derived from alveolar epithelial cells. These signals promote expression of the transcription factor PPAR-γ, which supports AM maturation (16, 17) and induces lipid-metabolism programs required for surfactant clearance and maintenance of local immune quiescence (16). Once established, AMs are maintained largely through *in situ* proliferation, with limited replenishment from peripheral blood monocytes under steady-state conditions (14, 15).

In experimental mouse studies, microbiota-related signals have also been reported to modulate AM homeostasis through TLR2/4-dependent upregulation of EI24. This pathway was associated with reduced AM apoptosis and restrained pro-inflammatory activation, although the extent to which comparable microbiota-dependent regulation shapes healthy human AMs remains to be clarified (18).

### 2.2 Interstitial macrophages: origins, maintenance, and niche-defined subsets

Unlike AMs in the alveolar lumen, IMs reside within the pulmonary interstitium near airways, blood vessels, and nerve bundles, where they comprise heterogeneous subsets with distinct origins, turnover dynamics, marker profiles, and functional programs. scRNA-seq profiling combined with kinetic studies has shown that the homeostatic IM compartment contains both early embryonic-derived resident cells and populations progressively replaced by bone marrow-derived monocytes with age. Under homeostatic or stress conditions, circulating monocytes recruited to the pulmonary interstitium can receive local niche-derived cues and differentiate into transcriptionally and functionally distinct IM subsets (19, 20).

One major subset occupies the perivascular niche around large blood vessels and is characterized by a LYVE1^+^CD206^+^ phenotype. This population shows relatively long-term persistence and local self-renewal capacity, and has been linked to vascular homeostasis and limitation of fibrotic remodeling, in part through expression of immunoregulatory factors such as IL-10 (19). Another subset occupies perineural and alveolar interstitial niches and is characterized by an MHCII^hi^CD206^−^ phenotype. This population expresses antigen-processing and presentation genes, including MHC-II–associated genes such as H2-Aa and Cd74 in mice, consistent with a role in immune surveillance within deeper lung tissues (19, 20).

Single-cell trajectory and kinetic studies further suggest that IMs may be replenished through multiple monocyte-derived routes. In murine models, classical Ly-6C^hi^ inflammatory monocytes contribute to the IM compartment, while non-classical Ly-6C^lo^ patrolling monocytes may also undergo extravasation and transition through a transient CD64^+^CD16.2^+^ intermediate state before differentiating into CD206^−^ IMs within the alveolar interstitium (20). Because murine Ly-6C^hi^ and Ly-6C^lo^ monocytes are often compared with human CD14^+^CD16^−^ and CD16^+^ monocyte populations, respectively, but are not exact equivalents, these replenishment pathways should be interpreted with attention to species context.

### 2.3 Pulmonary intravascular macrophages: vascular localization and species-specific features

PIMs represent a vascular macrophage population positioned along the pulmonary capillary endothelium, where they are suited to monitor blood-borne signals within the lung circulation. Their transcriptional programs are enriched for complement activation and iron handling, consistent with roles in blood-borne pathogen clearance and erythrocyte or iron homeostasis.

Cross-species scRNA-seq comparisons have suggested that the tissue localization and differentiation trajectories of non-classical monocytes differ between mice and humans. In mice, non-classical Ly-6C^lo^ monocytes have been linked mainly to IM replenishment (20). In human lung datasets, classical CD14^+^CD16^−^ monocytes were inferred to pass through an HLA-DR^hi^ transitional state after extravasation and contribute to AM and IM compartments, whereas non-classical CD16^+^ monocytes were found predominantly associated with the pulmonary capillary endothelium and were proposed to give rise to a PIM population characterized by high expression of complement genes, including C1QA/B/C, and iron metabolism-related genes, including CD163 and HMOX1 (21). These differences highlight the need to consider species context when interpreting PIM biology.

### 2.4 Molecular regulation of macrophage plasticity: epigenetic and metabolic reprogramming

While ontogeny and anatomical niche establish the broad organization of pulmonary macrophage subsets, their stable identity and context-dependent plasticity are further shaped by epigenetic and metabolic programs.

Single-cell assay for transposase-accessible chromatin sequencing (scATAC-seq) indicates that microenvironmental signals can influence macrophage response potential by remodeling chromatin accessibility. Under homeostatic conditions, AMs and IMs display distinct chromatin landscapes at transcription factor binding sites, which are associated with different transcriptional responses to external stimuli such as Toll-like receptor (TLR) ligands (22). Epigenomic profiling is particularly informative when macrophages with different origins acquire similar transcriptional profiles. In a murine severe influenza infection model, depletion of tissue-resident AMs was followed by recruitment of peripheral monocytes into the alveolar space and their differentiation into monocyte-derived alveolar macrophages (Mo-AMs). Although these Mo-AMs gradually converged with embryonic-derived AMs at the transcriptional level, scATAC-seq indicated that they retained chromatin features related to their monocytic origin. This “epigenetic memory” was associated with enhanced potential for IL-6 secretion and improved antibacterial protection upon secondary infection in this model (23).

**Figure 1:**
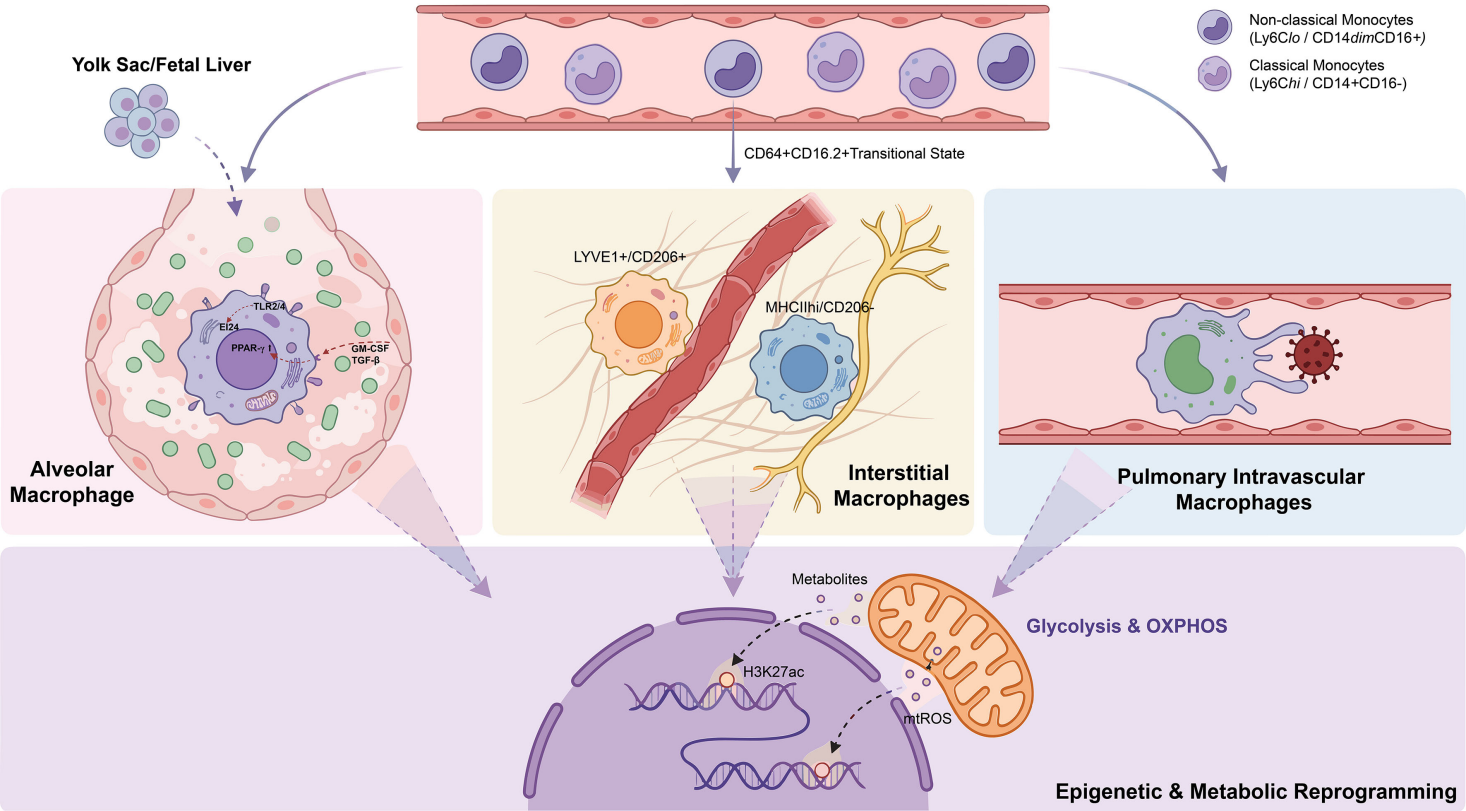
Homeostatic pulmonary macrophage compartments and regulatory programs. Pulmonary macrophages comprise anatomically and functionally distinct populations, including alveolar macrophages (AMs), interstitial macrophages (IMs), and pulmonary intravascular macrophages (PIMs). AMs reside in the alveolar airspace and are shaped by niche-derived signals such as GM-CSF and TGF-β, which promote PPAR-γ-dependent maturation and surfactant-handling programs. A microbiota-associated TLR2/4–EI24 pathway has also been reported to regulate AM homeostasis in mouse studies. IMs occupy perivascular, perineural, and alveolar interstitial niches and include resident and monocyte-replenished subsets, such as LYVE1^+^CD206^+^ perivascular IMs and MHCII^hi^CD206^−^ IMs. PIMs localize along the pulmonary vascular compartment and participate in vascular immune surveillance. Across these compartments, ontogeny, niche-derived cues, metabolic state, chromatin accessibility, and histone modifications jointly shape macrophage identity and response potential. AM, alveolar macrophage; IM, interstitial macrophage; PIM, pulmonary intravascular macrophage; GM-CSF, granulocyte–macrophage colony-stimulating factor; TGF-β, transforming growth factor-β; OXPHOS, oxidative phosphorylation; mtROS, mitochondrial reactive oxygen species.

Metabolic reprogramming is closely connected to epigenetic regulation because specific metabolic intermediates can regulate enzymes involved in chromatin and DNA modification. The EI24 pathway provides an example of such metabolic–epigenetic coupling in AMs. In experimental mouse studies, microbiota-related TLR2/4 signaling has been reported to maintain EI24 expression under homeostatic conditions. Loss or reduction of EI24 activity was associated with increased glycolysis and mitochondrial oxidative phosphorylation (OXPHOS), accompanied by accumulation of mitochondrial reactive oxygen species (mtROS). This metabolic shift was linked to broader changes in chromatin accessibility, including increased accessibility at binding sites of pro-inflammatory transcription factors such as AP-1. Through this metabolism–chromatin regulatory pathway, AMs may become more permissive to inflammatory activation and acquire a host-defense-associated phenotype (18) (**Figure 1**).

## 3 Macrophage diversity and function across distinct pulmonary pathologies

### 3.1 Chronic obstructive pulmonary disease

Chronic obstructive pulmonary disease (COPD) is characterized by chronic airway inflammation, emphysematous tissue destruction, and irreversible airflow limitation. Chronic exposure to cigarette smoke and other inhaled particulates is associated with oxidative stress, accumulation of macrophages and neutrophils, protease-antiprotease imbalance, and parenchymal destruction (24–26). Within this inflammatory and tissue-destructive microenvironment, macrophages contribute to mediator production, chemokine signaling, altered host defense, and tissue remodeling (27). Earlier bulk transcriptomic studies provided population-averaged views of macrophage activation, whereas single-cell studies of human COPD lung have revealed diverse macrophage transcriptional states within the alveolar niche. Rather than conforming to a single pro-inflammatory polarized state, COPD-associated macrophages display stress-response, inflammatory, chemokine, lipid-metabolic, and remodeling-related programs whose relative prominence varies across disease severity, tissue compartment, and sampling strategy (28–30).

#### 3.1.1 Exposure-associated oxidative stress and stress-response programs

In COPD lungs exposed to cigarette smoke-related stressors, oxidants and metal-containing particulates can perturb macrophage redox and metabolic homeostasis. In response, AMs may induce antioxidant and metal-detoxification programs. In human lung scRNA-seq analysis, Sauler et al. identified an advanced COPD-enriched AM subpopulation with high expression of metallothioneins, including MT1G, MT1X, and MT2A, together with increased expression of the antioxidant-response gene HMOX1. The study further validated increased MT2A expression in CD68^+^ phagocytes in COPD lung tissue (28). These findings support the presence of stress-response and metal-detoxification programs in COPD macrophages and suggest that such programs represent macrophage adaptation to oxidative and metal-associated injury within the COPD alveolar niche.

#### 3.1.2 Inflammatory, chemokine, and tissue-remodeling programs

With persistent injurious exposure, COPD macrophages display transcriptional programs linked to inflammatory signaling, chemokine production, and tissue remodeling. Rather than representing a uniform pro-inflammatory state, these programs reflect distinct but overlapping macrophage responses within the COPD lung microenvironment.

Transcriptomic studies have identified macrophage programs enriched for inflammatory and chemokine-related mediators. Sauler et al. reported increased expression of THBS1, PELI1, and CDC42 in COPD macrophages, with differentially expressed genes enriched in chemotaxis and inflammation (28). Fujii et al., using RNA-seq and lipidomic profiling of sorted bronchoalveolar lavage fluid (BALF) AMs, further showed GOLD grade-dependent AM transcriptional reprogramming, including lipid-metabolism changes and increased expression of chemokines such as CCL2, CCL8, and CCL20 in GOLD3/4 COPD (31). Together, these findings suggest that COPD macrophages acquire inflammatory and chemokine-response programs that may contribute to local immune-cell recruitment and inflammatory amplification, although functional and spatial validation is needed to define the underlying cellular interactions.

Macrophage–neutrophil interactions may further reinforce this inflammatory milieu. In experimental COPD-related models, CLEC5A^+^ macrophages have been linked to neutrophil activation and neutrophil extracellular trap formation, suggesting a macrophage–neutrophil interaction pathway that may contribute to airway inflammation (32).

In addition to inflammatory signaling, spatially resolved studies have begun to define macrophage programs associated with tissue remodeling in COPD. Spatial transcriptomic analyses have described disease-associated cellular communities in COPD lung tissue, in which immune, stromal, and structural cells are organized within pathological tissue neighborhoods (29). Consistent with this spatial organization, spatial multi-omics analysis identified a CHIT1^+^ remodeling-type IM population enriched for CHIT1, MMP9, and SPP1 within immune-rich interstitial niches. This population showed spatial association with lymphoid aggregates and regions of tissue destruction (30). These findings link CHIT1^+^ remodeling-type IMs to ECM remodeling and tissue injury in COPD, supporting a role for macrophage–stromal–immune interactions in the destructive lung microenvironment.

#### 3.1.3 Impaired host defense and ferroptosis-related signatures

In advanced or severe COPD contexts, macrophages may exhibit impaired host-defense programs alongside persistent inflammatory activation. As reviewed previously, AM dysfunction in COPD includes altered phagocytosis, defective efferocytosis, impaired microbial clearance, and persistent inflammatory activation (27, 33). Consistent with altered immune-surveillance programs, transcriptomic profiling of sorted BALF AMs has reported reduced expression of antigen-presentation-associated genes, including HLA-DQA2 and CD1B, together with changes in macrophage activation signatures and immune-related gene programs (31). Additional transcriptomic studies have described changes in macrophage subsets such as CD53-expressing macrophages, suggesting altered macrophage interactions with adaptive immune pathways (34).

At the molecular pathway level, lipid peroxidation and ferroptosis-related signatures have been proposed as candidate features of COPD macrophage dysfunction. Integrated scRNA-seq and transcriptomic analyses of COPD bronchoalveolar lavage fluid reported enrichment of ferroptosis-associated genes, including increased ALOX5 and CYBB and reduced GPX4, HSPB1, and SOCS1 (35). These findings identify ferroptosis-related transcriptional remodeling as a component of COPD-associated macrophage dysfunction rather than an established driver of emphysema progression (**Table 1**).

**Table 1:** Summary of macrophage subsets and functional profiles in the COPD microenvironment.

COPD-associated context	Subset/state	Representative markers/genes	Location/niche	Evidence-supported function/key pathway
Oxidative stress response	Metallothionein-high stress-response AMs	MT1G, MT1X, MT2A, HMOX1	Alveolar/distal airway	Stress-response and metal-detoxification program associated with cigarette smoke-related oxidative and metal exposure (28).
Inflammatory chemokine response	Chemokine-enriched inflammatory macrophage state	THBS1, PELI1, CDC42, CCL2, CCL8, CCL20	Inflamed airway/interstitium	Inflammatory and chemokine-related transcriptional program linked to immune-cell recruitment and local inflammatory amplification (28, 31).
Innate inflammatory effector response	CLEC5A-associated inflammatory macrophages	CLEC5A, TNF-α, IL-6, MMP12	Injured airway/alveolar compartment	CLEC5A-mediated macrophage activation enhances responsiveness to inflammatory stimuli and contributes to CS-induced inflammation, cytokine production, MMP12 expression, and airspace enlargement in experimental models (32, 34).
Tissue remodeling	CHIT1^+^ remodeling IMs	CHIT1, MMP9, SPP1, CHI3L1	Immune-rich interstitium	Spatially associated with lymphoid aggregates, ECM remodeling programs, and regions of tissue destruction in COPD lung (30).
Impaired immune surveillance	Antigen-presentation-low AM state	HLA-DQA2, CD1B	Alveolar/parenchymal injury regions	Reduced antigen-presentation signatures and altered immune-surveillance programs in COPD AMs (31).
Ferroptosis-related transcriptional remodeling	Ferroptosis-related macrophage signature	ALOX5, CYBB, GPX4, HSPB1, SOCS1	Alveolar macrophage compartment	Ferroptosis-related signature suggesting increased lipid-peroxidation stress and reduced anti-ferroptotic defense in COPD macrophages (35).

### 3.2 Asthma

Asthma is a heterogeneous respiratory disorder characterized by chronic airway inflammation, variable and often reversible airflow limitation, airway hyperresponsiveness, and progressive airway remodeling. In many asthma phenotypes, allergen exposure and epithelial-derived inflammatory cues initiate immune activation. During this process, diverse inflammatory cells—including eosinophils, mast cells, lymphocytes, and macrophages—interact through cytokine and chemokine networks to sustain airway inflammation (36). As abundant resident immune cells in the lung, macrophages exhibit substantial diversity and functional heterogeneity within the asthmatic microenvironment. Earlier murine allergen-challenge studies suggested divergent roles for resident and recruited macrophage populations: resident alveolar macrophages may limit allergic inflammation, whereas recruited monocyte-derived macrophages can promote airway inflammation and remodeling, including through epithelial CCL2-dependent recruitment pathways (37, 38). Although these experimental models provide useful mechanistic insight, their relevance to human macrophage states requires further validation. Recent human BALF scRNA-seq and experimental single-cell studies further support substantial macrophage remodeling in asthmatic microenvironments (39–41).

#### 3.2.1 Acute exacerbation and inflammatory chemotaxis

During acute exacerbation or allergen-driven inflammatory settings, pulmonary macrophages can adopt inflammatory and chemotaxis-associated transcriptional states. In a 16-week HDM-induced mouse model of chronic asthma, scRNA-seq analysis identified an alveolar macrophage subpopulation with increased expression of chemokine- and neutrophil-response-related transcripts, including murine Cxcl15, Ccl6, Ccl9, and Csf3r. This profile defines an inflammatory chemotactic macrophage state within the remodeled airway microenvironment (41).

Human BALF scRNA-seq studies of acute asthma exacerbation similarly reported expansion of monocytes and macrophages, together with remodeling of resident macrophage subpopulations in the pulmonary immune compartment. These macrophage populations expressed inflammation-associated transcription factors, including STAT1 and SPI1, as well as cytokine and chemokine genes such as IL1B, IL18, TNF, and CCL2, supporting their involvement in local inflammatory amplification and immune-cell recruitment during exacerbation (39). In severe or steroid-resistant asthma contexts, scRNA-seq has further revealed broad immune-cell remodeling under corticosteroid treatment. For example, in an HDM/LPS-induced steroid-resistant mouse model, inflammatory immune-cell states persisted despite glucocorticoid exposure, highlighting multicellular immune remodeling as a feature of refractory exacerbation phenotypes (42). Together, these findings indicate that macrophage plasticity contributes not only to allergen-associated airway inflammation but also to macrophage-associated immune programs observed in exacerbation and refractory disease contexts.

#### 3.2.2 Compartmental remodeling and compensatory immunoregulatory states

As asthma progresses into chronic remodeling contexts, macrophage compartment composition can shift, including changes in the balance between AMs and interstitial macrophage-like populations. In the chronic HDM-induced mouse model described above, scRNA-seq analysis showed expansion of interstitial macrophage-like cells and selected alveolar macrophage subsets within the asthmatic lung (41). These expanded interstitial macrophage-like cells did not uniformly display a pro-inflammatory profile. Instead, they showed reduced expression of neutrophil/granulocyte chemotaxis-related transcripts and relative enrichment of immune-defense or regulatory genes, including murine Il1b and Slpi, suggesting a context-dependent immunoregulatory program within the chronically inflamed airway microenvironment (41). Concurrently, proliferating AM subsets expressing cell-cycle genes such as Top2a and Mki67 may reflect local macrophage pool maintenance or repair-associated responses to chronic epithelial injury (41).

#### 3.2.3 Impaired resolution of inflammation and airway remodeling

After allergen clearance, macrophages can contribute to inflammation resolution and tissue repair. In chronic or recurrent asthma, however, resolution may be incomplete, and macrophages can participate in persistent interactions with epithelial, stromal, and immune cells. These interactions are associated with airway remodeling, including airway smooth muscle changes, basement membrane thickening, extracellular matrix deposition, and subepithelial fibrosis (43–46).

Single-cell and cell–cell communication analyses in a chronic HDM-induced mouse model identified strengthened macrophage–fibroblast interactions in the remodeled asthmatic lung, although many ligand–receptor relationships remain computational predictions requiring spatial and functional validation (41). In this model, macrophage subsets were inferred to communicate with Col14a1^+^ fibroblast/myofibroblast populations through IL1B-, TGFB1-, and SPP1-related signaling, linking macrophage inflammatory programs with fibroblast activation and remodeling-associated gene expression (41). Reciprocally, Col14a1^+^ fibroblast populations expressed stromal and chemotactic mediators, including Postn, Igf1, Ccl8, and Cxcl12, which may influence macrophage recruitment, activation, or stromal–immune communication (41). This framework supports a bidirectional macrophage–stromal axis in asthma remodeling while emphasizing the need for validation in human asthma tissues and spatially resolved datasets (**Table 2**).

**Table 2:** Summary of macrophage subsets and functional profiles in the asthma microenvironment.

Asthma-associated context	Subset/state	Representative markers/genes	Location/niche	Evidence-supported function/key pathway
Acute exacerbation	Inflammatory chemokine-high macrophages	STAT1, SPI1, IL1B, IL18, TNF, CCL2	Human BALF/Inflamed airway	Inflammatory amplification and immune-cell recruitment during acute exacerbation (39).
Chronic inflammatory remodeling	Chemotactic/neutrophil-response AM state	Cxcl15, Ccl6, Ccl9, Csf3r	Alveolar macrophage compartment in HDM-induced mouse lung	Chemokine signaling and neutrophil-response programs in chronic airway inflammation (41).
Chronic phase	Regulatory-like IM state	Il1b, Slpi	Interstitial macrophage-like compartment in HDM-induced mouse lung	Compensatory immune-defense and regulatory adaptation during chronic inflammation (41).
Chronic repair	Proliferating AM state	Top2a, Mki67	Alveolar macrophage compartment in HDM-induced mouse lung	Local macrophage pool maintenance and repair-associated response to epithelial injury (41).
Airway remodeling	Fibroblast-interacting macrophage state	Il1b, Tgfb1, Spp1	Stromal–immune interface in remodeled HDM-induced mouse lung	Macrophage–fibroblast communication, fibroblast activation, and ECM remodeling (41).

### 3.3 Pulmonary fibrosis

Pulmonary fibrosis encompasses a spectrum of interstitial lung diseases, including idiopathic pulmonary fibrosis and other progressive fibrotic disorders, that are characterized by excessive extracellular matrix (ECM) deposition, interstitial scarring, and distortion of alveolar architecture (47). Although activated fibroblasts and myofibroblasts are the main effector cells responsible for ECM production, fibrotic remodeling arises from coordinated interactions among epithelial, endothelial, immune, and stromal compartments. Within this multicellular microenvironment, macrophages act as important regulators of fibrotic niche remodeling by linking injury sensing and inflammatory signaling to fibroblast activation, epithelial repair, and barrier remodeling (48–50).

#### 3.3.1 Homeostatic disruption and transitional macrophage programs

In normal lung tissue, resident macrophages express genes such as PPARG, MRC1, and MARCO and contribute to immune homeostasis (51). During fibrotic remodeling, recruited monocytes and local macrophage adaptation reshape the macrophage pool. Spatial transcriptomic analysis of pulmonary fibrosis samples indicates that FABP4^+^ macrophages are enriched in injured airspaces within early remodeling regions before overt alveolar destruction, possibly reflecting debris clearance and stress adaptation (52). These observations support a spatial association with regions classified as early remodeling but do not establish direct transdifferentiation into later SPP1-associated macrophage subsets.

In a bleomycin-induced mouse fibrosis model, trajectory and reference-based analyses identified CX3CR1^+^SiglecF^+^ transitional macrophages along a monocyte-to-macrophage continuum in injured lung tissue (53). These cells expressed mediators such as PDGF-AA and localized to fibrotic niches, supporting a role in fibroblast recruitment and proliferation in this experimental setting. Orthologous signatures of these transitional macrophages were also detected in human IPF samples, suggesting partial relevance to human fibrotic disease, although direct lineage and functional validation in human lung tissue remain needed (53).

#### 3.3.2 SPP1-associated macrophages and fibroblast-rich fibrotic niches

In active remodeling regions of fibrotic lung, macrophage populations expressing SPP1 and remodeling-associated genes such as CHI3L1, MMP9, and MERTK become enriched (51, 52). Their transcriptional profiles are associated with exocytosis, ECM organization, and cell-migration pathways, consistent with participation in fibrotic niche remodeling (51).

Spatial studies further show that SPP1-expressing macrophages are frequently located near fibroblastic foci and fibroblast-rich regions. Through mediators and pathways involving SPP1, TGF-β-related signaling, and MMP9, these macrophages are linked to fibroblast activation and ECM remodeling (51, 52). In fibrotic regions, SPP1^+^/MERTK^+^ macrophages also show increased proliferative indices compared with normal lung, and CSF1-related signaling has been implicated in their maintenance (51, 54). These findings suggest that fibroblast-rich macrophage niches may be sustained by both monocyte recruitment and local macrophage persistence, although their relative contributions remain unresolved.

In connective tissue disease-associated interstitial lung disease, spatial transcriptomics has identified macrophage-dominated niches that include multinucleated giant cells, consistent with persistent inflammation and remodeling in selected fibrotic lesions (55).

#### 3.3.3 Macrophage interactions with barrier and regenerative niches

Beyond their interactions with fibroblasts, macrophages may influence fibrotic remodeling through crosstalk with endothelial and epithelial compartments. In experimental fibrosis-related injury, macrophage-derived MMP12 has been shown to promote endothelial dysfunction and vascular leak, providing a candidate macrophage–endothelial mechanism relevant to fibrotic progression (56).

Macrophage–epithelial interactions are also increasingly recognized in human IPF tissue. CD206/MRC1-associated macrophage populations have been spatially mapped near aberrant basaloid cells (ABCs), supporting a candidate interaction between macrophages and abnormal epithelial repair niches (57). These observations are consistent with impaired alveolar regeneration in IPF, but they should be interpreted as spatial associations and candidate mechanisms rather than definitive proof that macrophages directly cause epithelial regeneration arrest.

Spatial multi-omics studies of progressive and reversible fibrotic remodeling further indicate that fibroblast heterogeneity may shape macrophage behavior. Macrophages can localize near ECM-producing fibroblast populations, but may also coexist with fibroblast states associated with anti-fibrotic or pro-resolving programs in selected niches (58). This suggests that macrophage function in fibrosis is context-dependent and may include both injury-amplifying and resolution-associated roles (**Table 3**).

**Table 3:** Summary of macrophage subsets and functional profiles in the pulmonary fibrosis microenvironment.

Pulmonary fibrosis-associated context	Subset/state	Representative markers/genes	Location/niche	Evidence-supported function/key pathway
Early airspace remodeling	FABP4^+^ airspace macrophages	FABP4	Injured airspaces/early-remodeling regions	Early spatial accumulation, debris clearance, and stress adaptation (52).
Monocyte-to-macrophage transition	CX3CR1^+^SiglecF^+^ transitional macrophages	CX3CR1, SiglecF, PDGF-AA	Injured interstitium/fibrotic niche in experimental fibrosis	Trajectory-inferred transitional state linked to fibroblast recruitment and niche priming (53).
Fibroblast-rich remodeling niche	SPP1-associated remodeling macrophages	SPP1, CHI3L1, MMP9, MERTK	Fibroblast-rich regions/fibroblastic foci	Fibroblast activation, ECM remodeling, and CSF1-related macrophage persistence (51, 52, 54).
Vascular barrier injury	MMP12-expressing macrophages	MMP12	Endothelial injury regions/experimental fibrosis-related injury	Endothelial barrier dysfunction and vascular leak in experimental injury (56).
Aberrant epithelial repair	MRC1/CD206-associated macrophages	MRC1/CD206	ABC-associated regenerative niches in IPF tissue	Spatial association with aberrant basaloid cells and impaired alveolar repair (57).

### 3.4 Lung cancer

The implementation of low-dose computed tomography screening has improved the detection of early-stage lung cancer (59), yet advanced disease remains difficult to treat because many patients develop primary or acquired resistance to chemotherapy, targeted therapy, or immunotherapy (60, 61). Multi-omics studies have broadened the focus from the tumor–T cell axis alone to the wider tumor immune microenvironment. Within this ecosystem, tumor-associated macrophages (TAMs) are highly plastic myeloid populations that link tumor genetics, host inflammation, stromal remodeling, and therapeutic response (62–65).

#### 3.4.1 Resident macrophages and early microenvironmental remodeling

TME remodeling can begin during premalignant and carcinoma *in situ* stages. Single-cell trajectory analyses suggest that tissue-resident macrophages, including alveolar macrophage-like populations, are among the early responders that localize near aberrantly proliferating epithelial cells (66, 67). In experimental KRAS-driven lung cancer, macrophage subsets with senescence-associated secretory features express chemokines such as CCL2 and CXCL13, supporting their involvement in early immune-cell recruitment and microenvironmental remodeling (68).

#### 3.4.2 TAM subset remodeling during tumor invasion and progression

As lesions progress, the TAM compartment undergoes substantial remodeling. CXCL9^+^ TAM programs, which are generally associated with interferon-response and anti-tumor immune activity, decline in some datasets, whereas TREM2^+^ and SPP1^+^ TAMs expand and are linked to angiogenesis, matrix remodeling, and invasive phenotypes (69). Similar immune-state transitions have also been described in analyses of part-solid nodules, which may represent more invasive early lung adenocarcinoma states (70, 71).

Beyond changes in subset abundance, TAM function is shaped by epigenetic and metabolic reprogramming. A TET3-high macrophage population has been associated with increased expression of NLRP3, IL1B, and CD274 in lung cancer (72). Other studies have identified phagocytic TAM states that display increased oxidative phosphorylation and upregulation of CD73 and PD-L1 after tumor-cell engulfment, linking phagocytic activity to metabolic adaptation, immune suppression, and adverse clinical outcomes in selected cohorts (73, 74).

#### 3.4.3 Specialized TAM programs under advanced disease and therapeutic pressure

In invasive adenocarcinoma, immunoregulatory TAM programs become more prominent. Across tumor types, C1Q^+^ TAM programs have been described as macrophage states frequently expressing C1QA/B/C together with APOE, MRC1/CD206, HLA-DR, and, in some contexts, FOLR2 or TREM2. These programs have been associated with T cell exhaustion, immune tolerance, and poor prognosis in several cancers, as reviewed previously, while their direct functional role in NSCLC remains incompletely defined (71, 75).

Under therapeutic pressure, macrophage composition may further reorganize. In a small scRNA-seq/scTCR-seq study of rare-driver-mutated NSCLC after anti-PD-1 agents combined with chemotherapy, non-MPR tumors showed enrichment of FABP4^+^ and CHIT1^+^ macrophages and higher M2-associated signatures. OAS1 expression in FABP4^+^ and CHIT1^+^ macrophages was supported by multiplex immunofluorescence, and CellChat analysis nominated malignant-cell interactions with macrophages through LAMC2–ITGA6/ITGB1 as a candidate communication axis associated with non-response (76). Because this cohort was small and lacked paired longitudinal baseline samples, these findings should be interpreted as therapy-associated macrophage remodeling rather than definitive evidence of macrophage-driven resistance.

#### 3.4.4 Systemic myeloid programming and bone marrow-level regulation

The pro-tumor characteristics of TAMs are shaped not only by the local TME but also by systemic myeloid programming. In experimental lung cancer, tumor-derived signals can induce basophils and eosinophils in the bone marrow to produce IL-4, which acts on granulocyte–monocyte progenitors and biases subsequent macrophage differentiation toward tumor-supportive states (77). These findings expand the spatial scale of TAM regulation from the local TME to bone marrow-level myeloid programming (**Table 4**).

**Table 4:** Summary of macrophage subsets and functional profiles in the lung cancer microenvironment.

Lung cancer-associated context	Subset/state	Representative markers/genes	Location/niche	Evidence-supported function/key pathway
Early microenvironmental remodeling	Resident macrophage-associated early remodeling state	CCL2, CXCL13	Peri-epithelial premalignant niche	Early immune-cell recruitment and premalignant niche remodeling (66–68).
Tumor invasion and progression	TREM2^+^/SPP1^+^ remodeling TAMs	TREM2, SPP1	Tumor stroma/invasive tumor regions	Associated with angiogenesis, matrix remodeling, and invasive tumor programs (69, 70).
Epigenetic immunoregulation	TET3-high immunoregulatory TAMs	TET3, NLRP3, IL1B, CD274	Tumor inflammatory niches	Linked to IL-1β-related inflammatory signaling and checkpoint-associated immunoregulation (72).
Phagocytic metabolic adaptation	OXPHOS-high phagocytic TAMs	NT5E, COX4I1, ATP5F1A, NDUFA4	Tumor parenchyma/phagocytic TAM compartment	Post-phagocytic metabolic adaptation and adenosine/checkpoint-associated immunosuppression (73, 74).
Advanced immune regulation	C1Q^+^ immunoregulatory TAM programs	C1QA, C1QB, C1QC, APOE, MRC1, HLA-DRA	Tumor immune niches	Associated with T cell exhaustion-related and immune-tolerant programs across tumor types (71, 75).
Therapy-associated non-response	Non-MPR-enriched FABP4^+^/CHIT1^+^ macrophage states	FABP4, CHIT1, OAS1, MRC1, CD163	Post-treatment rare-driver-mutated NSCLC TME	Enriched in non-MPR tumors after anti-PD-1 plus chemotherapy; LAMC2–ITGA6/ITGB1 nominated as a candidate malignant cell–macrophage interaction axis (76).

### 3.5 Pulmonary infection and lung injury

Severe pulmonary infection, acute lung injury, and acute respiratory distress syndrome (ARDS) remain major challenges in respiratory critical care. Protective host defense requires pathogen clearance followed by timely resolution of inflammation and tissue repair (78). When inflammatory responses are excessive or poorly resolved, alveolar–capillary barrier dysfunction, edema, and respiratory failure can occur (79–81).

Neutrophils are prominent contributors to acute lung injury (78). However, single-cell and multi-omics studies have shown that AM loss or reduction, monocyte recruitment, and macrophage reprogramming are also central features of severe lung injury and repair. Across infection- and injury-related contexts, macrophage states span a continuum from inflammatory amplification to resolution and epithelial regeneration (82).

#### 3.5.1 Inflammatory amplification, barrier dysfunction, and immune crosstalk

During severe pulmonary infection, including severe COVID-19, and in sepsis-associated acute lung injury, resident AMs can be reduced while circulating monocytes are recruited into the lung. In BALF from patients with severe COVID-19, FCN1^+^ monocyte-derived macrophages were enriched and expressed chemokines such as CCL2 and CCL3, together with cytokines including IL1B and IL6 (83). These findings support a macrophage-associated inflammatory program within the injured alveolar compartment, but do not establish macrophages as the sole driver of systemic hyperinflammation.

Inflammatory macrophage programs can also influence barrier cells and neutrophil responses. In sepsis-associated acute lung injury, IL-1β-expressing lung macrophages have been linked to endothelial dysfunction through p38 MAPK signaling, supporting a macrophage–endothelial pathway contributing to barrier injury (84). In radiation-induced lung injury datasets, integrative transcriptomic and single-cell analyses suggested bidirectional crosstalk between NETosis-associated neutrophil programs and macrophage responses, including SPP1–CD44, ANXA1–FPR, and CCL/CXCL signaling axes (85). Together, these findings support a broader framework in which macrophage–endothelial and macrophage–neutrophil interactions may contribute to tissue injury, particularly through inflammatory cytokine, chemokine, and damage-associated signaling pathways.

#### 3.5.2 Macrophage reprogramming, epithelial repair, and post-injury memory

Resolution and repair depend in part on whether macrophages transition from inflammatory states toward programs that support epithelial regeneration. During recovery from H1N1 influenza infection, monocyte-derived alveolar macrophages (Mo-AMs) accumulate near regenerative foci and secrete oncostatin M (OSM), which activates JAK/STAT3 signaling in AT2 cells and supports AT2 proliferation and differentiation toward AT1 cells (86). Depletion experiments in this model support a functional role for these cells in endogenous alveolar repair.

Repair can become maladaptive when inflammatory and fibrotic programs persist. Spatial mapping of fatal COVID-19/ARDS lung tissue showed macrophage accumulation near differentiation-arrested transitional epithelial progenitor-like cells (DATPs), suggesting an association between macrophage-rich inflammatory or pro-fibrotic niches, impaired epithelial differentiation, lung consolidation, and fibrotic sequelae (87).

Acute injury can also leave persistent epigenetic imprints in macrophages. In a murine influenza model, repopulating alveolar macrophages that arise after severe viral injury regained many homeostatic transcriptional features months after recovery but retained altered chromatin accessibility. This stress imprint altered responses to secondary bacterial challenge, highlighting post-injury epigenetic memory as a mechanism by which macrophages may shape subsequent host responses (23). The persistence and clinical relevance of comparable macrophage memory programs in human survivors of severe pulmonary infection remain important areas for future study (**Table 5**).

**Table 5:** Summary of macrophage subsets and functional profiles in the pulmonary infection and lung injury microenvironment.

Pulmonary infection/lung injury context	Subset/state	Representative markers/genes	Location/niche	Evidence-supported function/key pathway
Severe infection/acute lung injury	FCN1^+^ inflammatory monocyte-derived macrophages	FCN1, IL1B, IL6, CCL2, CCL3	Injured alveolar compartment; perivascular or inflammatory niches	Contributes to local inflammatory amplification through IL1B/IL6 and CCL2/CCL3 expression; linked to endothelial barrier dysfunction and neutrophil-associated injury pathways in acute lung injury settings (83–85).
Regenerative repair	OSM^+^ reparative monocyte-derived alveolar macrophages	OSM, CTSB, CTSD, CTSS, CD74	AT2-adjacent regenerative foci	Supports AT2 proliferation and alveolar repair through OSM–JAK/STAT3 signaling during murine influenza recovery (86).
Aberrant repair/fibroproliferation	SPP1^+^ injury-associated macrophages	SPP1, TGFB1, TREM2, CCL18	DATP-associated or consolidated lung regions	Associated with impaired epithelial differentiation and fibrotic remodeling in severe COVID-19/ARDS lung tissue (87).
Post-injury memory	Stress-imprinted repopulating alveolar macrophages	SIGLECF, MERTK	Post-injury alveolar niche	Retains injury-induced chromatin remodeling and alters responses to secondary bacterial challenge in murine influenza models (23).

### 3.6 Pulmonary hypertension

Pulmonary hypertension (PH) is characterized by progressive remodeling of distal pulmonary arteries, including endothelial dysfunction, pulmonary arterial smooth muscle cell (PASMC) proliferation and muscularization, and extracellular matrix (ECM) accumulation (88, 89). Histological and single-cell studies indicate that perivascular inflammatory-cell infiltration may occur early during disease evolution and can precede or accompany overt vascular remodeling (90–92). These findings support a contribution of immune mechanisms, including monocyte/macrophage populations, to vascular remodeling in selected PH contexts (93, 94).

#### 3.6.1 Monocyte recruitment and perivascular macrophage niches

In pulmonary vascular disease models, remodeling-associated macrophage populations appear to include a substantial contribution from recruited circulating monocytes, whereas resident alveolar macrophages are less directly linked to perivascular remodeling (95). This distinction suggests that vascular macrophage states are shaped by systemic inflammatory cues as well as by local endothelial, smooth-muscle, and adventitial niches.

In response to hypoxia, endothelial injury, and altered shear stress, pulmonary vascular cells can produce mediators and upregulate chemokines, including CCL2, CCL5, and CX3CL1, that are associated with monocyte recruitment (96–99). After entering the perivascular compartment, recruited monocytes can adopt macrophage states enriched for inflammatory and remodeling-associated transcripts (100–102). Interactome analyses and experimental studies suggest that macrophage-associated signals, including thrombospondin-1, IL-1β, WNT11, and related pathways, may influence PASMC phenotypic switching, proliferation, and migration. Macrophages and adventitial fibroblasts may also participate in CSF1/CCL2-associated feedback circuits that sustain inflammatory remodeling of the vascular wall (103–105).

#### 3.6.2 Macrophage metabolic reprogramming and fibrotic remodeling

With persistent perivascular inflammation, vascular remodeling can progress toward fibroproliferative or obstructive lesions. In chronic thromboembolic pulmonary hypertension, macrophages within organizing thrombi have been reported to upregulate sphingosine kinase 1 (SPHK1), supporting TGF-β1 production and fibroblast Smad/STAT3 activation (106). This pathway provides a candidate mechanism for macrophage-associated thrombus fibrosis, but its relevance to other PH subtypes remains less clear (**Table 6**).

**Table 6:** Summary of macrophage subsets and functional profiles in the pulmonary hypertension microenvironment.

PH-associated context	Subset/state	Representative markers/genes	Location/niche	Evidence-supported function/key pathway
Early recruitment	Chemokine-responsive recruited monocyte/macrophage populations	CCR2, CCR5, CX3CR1	Perivascular and adventitial vascular niches	Supports chemokine-driven monocyte recruitment and perivascular macrophage accumulation (96–102).
Early remodeling	Remodeling-associated perivascular macrophages	THBS1, IL1B, WNT11	Perivascular and adventitial niches	May influence PASMC phenotypic switching, proliferation, and migration, and participate in macrophage–fibroblast CSF1/CCL2-associated remodeling circuits (103–105).
Late fibrotic remodeling	SPHK1^+^ thrombus-associated macrophages	SPHK1, TGFB1	Organizing thrombi in CTEPH	Links macrophage SPHK1 to TGF-β1 production and fibroblast Smad/STAT3 activation during thrombus fibrosis (106).

## 4 Cell–cell communication at single-cell resolution

**Figure 2:**
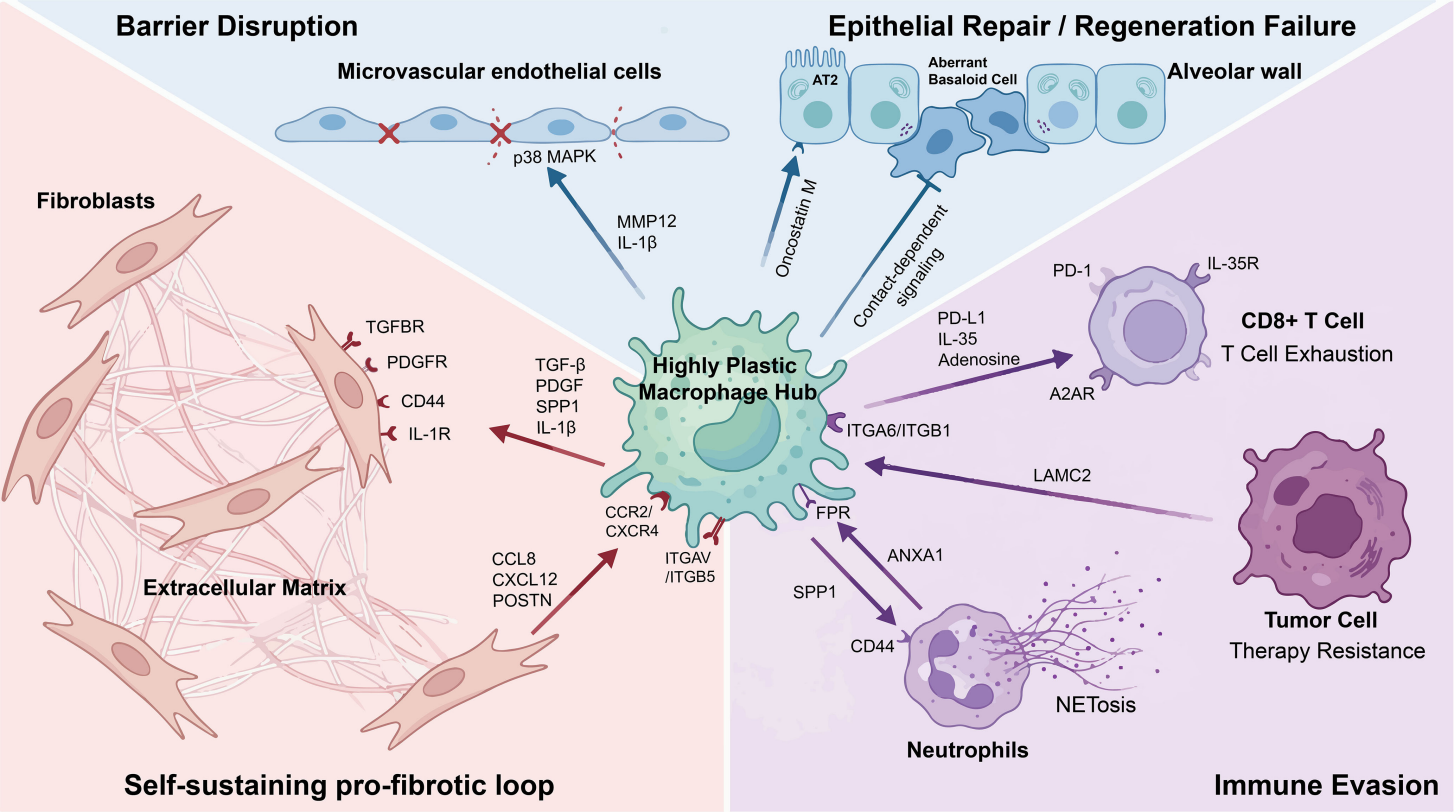
Macrophage-centered communication networks in pulmonary disease niches. Pulmonary macrophages can act as plastic signaling hubs that connect stromal, epithelial, endothelial, immune, and tumor-cell compartments. In remodeling niches, inflammatory or remodeling-associated macrophage states may express TGF-β-related ligands, PDGF-family signals, SPP1, and IL-1β, which are linked to fibroblast activation, myofibroblast-associated programs, and extracellular matrix remodeling. Fibroblast-derived mediators, including CCL8, CXCL12, and POSTN, may in turn support monocyte recruitment, macrophage activation, and stromal–immune communication. In barrier and repair niches, macrophage-derived IL-1β and MMP12 are associated with endothelial stress and barrier dysfunction, whereas OSM-producing macrophages can support alveolar epithelial repair in influenza recovery models. In severe injury or fibrotic contexts, macrophage-rich niches are spatially associated with aberrant epithelial repair states. In tumor or chronic inflammatory niches, macrophages may influence CD8^+^ T cell function through checkpoint, cytokine, and metabolic pathways, and ligand–receptor analyses nominate candidate macrophage–tumor or macrophage–neutrophil axes such as LAMC2–ITGA6/ITGB1, SPP1–CD44, and ANXA1–FPR. ECM, extracellular matrix; OSM, oncostatin M; TAM, tumor-associated macrophage.

The disease-specific sections above highlight recurrent macrophage states across inflammatory, fibrotic, vascular, and tumor niches. A complementary question is how these macrophages communicate with neighboring structural and immune cells. Conventional *in vitro* co-culture models provide controlled systems for testing selected cell–cell interactions, but they cannot fully recapitulate the spatial organization, cellular diversity, and niche-specific signaling of diseased lung tissue. Integration of scRNA-seq with spatial transcriptomic or proteomic approaches can prioritize ligand–receptor (L–R) expression patterns within tissue context and generate hypotheses about cell–cell communication. Because most L–R analyses rely on transcript abundance and curated interaction databases, they should be interpreted as hypothesis-generating evidence for potential signaling relationships. Confidence in these predicted interactions increases when they are supported by spatial co-localization, protein-level data, perturbation experiments, or clinical association (107). Across pulmonary diseases, macrophages frequently emerge as candidate signaling nodes that interact with fibroblasts, epithelial and endothelial cells, neutrophils, lymphocytes, and tumor cells (**Figure 2**).

### 4.1 Macrophage–fibroblast axis: candidate stromal remodeling networks

In COPD-associated airway remodeling, IPF, and PH, single-cell communication analyses frequently identify macrophage–fibroblast interactions as candidate networks associated with tissue remodeling (107). Recruited or inflammatory macrophage populations, including SPP1^+^ or TREM2^+^ subsets, can express TGF-β-related ligands, PDGF-family signals, SPP1, and IL-1β, which are predicted or, in selected models, experimentally supported to engage receptors such as TGFBR, PDGFRA, and CD44 on fibroblasts (51, 104). Conversely, fibroblast-derived mediators such as CCL8, CXCL12, and POSTN may promote monocyte recruitment, macrophage activation, or stromal–immune communication (41). Spatial transcriptomic studies further support close localization of macrophage and fibroblast populations in remodeling-associated structures such as fibroblastic foci (52).

### 4.2 Macrophage–barrier cell axis: barrier dysfunction and epithelial repair

The integrity of the alveolar–capillary barrier is essential for efficient gas exchange and fluid homeostasis. Aberrant macrophage signaling to endothelial or epithelial cells has been associated with barrier injury, impaired epithelial regeneration, or maladaptive repair. In acute injury and fibrosis-related settings, macrophage-derived IL-1β and MMP12 have been linked to endothelial stress signaling, tight-junction disruption, or vascular leak (56, 84).

During alveolar epithelial repair, macrophage-derived paracrine signals may influence progenitor-cell fate. In influenza recovery, OSM produced by monocyte-derived alveolar macrophages activates JAK/STAT3 signaling in AT2 cells and supports epithelial regeneration (86). In contrast, severe COVID-19/ARDS and IPF datasets show macrophage proximity to DATPs or aberrant basaloid cells, suggesting that macrophage-rich niches may be associated with failed epithelial differentiation and tissue scarring (57, 87).

### 4.3 Macrophage–immune axis: immune regulation and therapy-associated remodeling

In lung cancer and chronic inflammatory diseases, macrophages can modulate adaptive immune responses through checkpoint, cytokine, complement, and metabolic pathways. These programs are associated with local immune tolerance and may influence therapeutic response.

Single-cell and multi-omics analyses suggest that TAM-associated suppression of CD8^+^ T cells extends beyond the PD-L1/PD-1 checkpoint. Phagocytic TAM states have been linked to CD73 and PD-L1 induction after tumor-cell engulfment (73, 74), whereas C1Q^+^ TAM programs, as reviewed across tumor types, have been associated with complement-related signaling, EBI3/IL-35-related molecules, checkpoint-ligand expression, and T cell exhaustion-related features (75). These findings support a broader view of TAM-mediated immune regulation, while the relative contribution of each pathway in NSCLC remains context-dependent.

Under therapeutic pressure such as neoadjuvant immunotherapy, macrophage–tumor interaction networks may also be remodeled. In non-responsive lung cancer samples, malignant cells and macrophages showed predicted LAMC2–ITGA6/ITGB1 interactions associated with tumor-promoting macrophage states (76). In chronic lung injury, macrophage–neutrophil communication involving SPP1–CD44 and ANXA1-associated pathways has been proposed to sustain inflammatory circuits (85). Together, these examples illustrate how L–R analysis can nominate disease-relevant interaction axes, while orthogonal validation remains necessary before assigning causal roles.

## 5 Current limitations and unresolved questions

Although single-cell and spatial multi-omics have greatly expanded our understanding of pulmonary macrophage heterogeneity, several limitations should be considered when interpreting macrophage plasticity across disease contexts. Murine models remain indispensable for fate mapping, lineage tracing, and perturbation experiments, but macrophage ontogeny, niche adaptation, and monocyte-to-macrophage differentiation programs are not fully conserved between mice and humans. Findings from experimental models should therefore be interpreted alongside human lung tissue data, clinically relevant ex vivo systems, and spatial or proteomic validation (21, 107).

Trajectory-inference methods, including pseudotime, RNA velocity, and related computational approaches, are useful for ordering transcriptional states but do not by themselves prove lineage relationships or causal transitions. These analyses may be influenced by sampling time, tissue dissociation, cell-cycle effects, inflammatory intensity, and unmeasured niche signals. Similarly, defining stable macrophage states remains challenging because macrophage phenotypes are continuous, plastic, and shaped by tissue context. Marker-based labels such as SPP1^+^, TREM2^+^, CHIT1^+^, or FCN1^+^ are useful operational descriptors, but they may not define conserved cell types across diseases, species, platforms, or disease stages (108, 109).

Spatial transcriptomics provides essential tissue context, yet remains limited by capture efficiency, spatial resolution, transcript drop-out, incomplete protein-level information, and difficulty resolving cell boundaries in densely inflamed or fibrotic regions. Ligand–receptor analyses can nominate candidate communication axes, but functional signaling requires support from protein-level, spatial, downstream pathway, or perturbation-based evidence. Addressing these limitations through standardized nomenclature, shared reference atlases, multimodal validation, and longitudinal or interventional study designs will be essential for translating macrophage atlases into mechanistic and therapeutic insight (110–112).

## 6 Conclusions and future perspectives

Understanding the spatiotemporal plasticity of pulmonary macrophages is reshaping therapeutic concepts in lung disease. Broad macrophage depletion strategies, such as systemic CSF1R inhibition, may affect protective macrophage populations involved in host defense, tissue repair, and immune surveillance (113). Single-cell and spatial multi-omics therefore support a more selective framework focused on disease-associated macrophage states, niche-specific signals, and stage-dependent therapeutic windows (114).

For diseases characterized by matrix remodeling or immune suppression, including pulmonary fibrosis and lung cancer, therapeutic strategies targeting TREM2- or SPP1-associated macrophage programs, as well as macrophage–stromal signaling axes, are being explored (115). Macrophage-based and other CAR-engineered cellular therapies, together with engineered cytokine-delivery approaches, have also shown activity in preclinical solid-tumor models (116, 117). In hyperinflammatory lung injury, macrophage-targeted nanodelivery systems designed to modulate metabolic or epigenetic regulators may provide a preclinical strategy to reduce inflammatory signaling while preserving host defense, although clinical translation remains at an early stage (118).

Moving forward, integration of single-cell transcriptomics, spatial metabolomics, single-cell proteomics, and functional perturbation will be needed to construct more reliable spatiotemporal atlases of pulmonary disease. Computational methods and artificial intelligence may help identify macrophage-associated digital biomarkers, but such biomarkers must be validated across cohorts, species, platforms, and clinically meaningful endpoints. Ultimately, the therapeutic value of macrophage atlases will depend on whether disease-associated macrophage states can be linked to defined tissue niches, disease stages, functional mechanisms, and clinically actionable therapeutic windows.
